# Supplementary material for: Super Partition: fast, flexible, and interpretable large-scale data reduction in R
Source: PeerJ. 2025 Jan 27;13:e18580. doi: 10.7717/peerj.18580 (PMC11781262; doi:10.7717/peerj.18580)
Supplement: Supplemental Information 1 — This code includes a query to a portion of the data used for analysis as well as an example of how to complete the full analysis. [file peerj-13-18580-s001.pdf]

Super Partition: fast, flexible, and interpretable large-scale data reduction in R

April 22, 2024

1 Supplemental Material

Example Code

```
library(GEOquery)
library(partition)

# query CAMP gene expression data from GEO
## accession number - GSE22324
camp <- getGEO("GSE22324", GSEMatrix = TRUE)
dim(camp$GSE22324_series_matrix.txt.gz)

# get expression matrix
camp_ge <- as.data.frame(t(exprs(camp$GSE22324_series_matrix.txt.gz)))
dim(camp_ge)

# information loss criterion (ILC) values
ILClist <- seq(from=0, to=1, by=0.05)

# for each ILC
for (i in 1:length(ILClist)) {
  start.time <- Sys.time()

  # partition data at appropriate ILC
  camp_part <- super_partition(full_data = camp_ge,
                               threshold = ILClist[i],
                               cluster_size = 4000)

  # print time and number of features
  end.time <- Sys.time()
  diff <- difftime(end.time, start.time, units='mins')
  print(paste0("ILC = ", ILClist[i], " number of features: ",
               dim(camp_part$reduced_data)[[2]]))
  print(diff)
}
```
